# A modified oral sugar test for evaluation of insulin and glucose dynamics in horses

**DOI:** 10.1186/s13028-016-0246-z

**Published:** 2016-10-20

**Authors:** Sanna Lindåse, Katarina Nostell, Johan Bröjer

**Affiliations:** Department of Clinical Sciences, Swedish University of Agricultural Sciences, Box 7054, 750 07 Uppsala, Sweden

**Keywords:** Horse, Insulin dysregulation, Insulin resistance, Oral sugar test

## Abstract

**Background:**

An oral sugar test (OST) using Karo^®^ Light Corn Syrup has been developed in the USA as a field test for the assessment of insulin dysregulation in horses but the syrup is not available in Scandinavian grocery stores. The aim of the study was to compare the results of a modified OST between horses with equine metabolic syndrome (EMS) and healthy horses using a Scandinavian commercially available glucose syrup (Dansukker glykossirap). In addition, the effect of breed and the repeatability of the test were evaluated. In the present study, clinically healthy horses (7 Shetland ponies, 8 Icelandic horses, 8 Standardbred horses) and 20 horses of various breeds with EMS underwent the modified OST test. The Icelandic horses and Shetland ponies underwent the OST twice. Insulin and glucose data from the OST were used to calculate peak insulin concentration (Peak_INS_), time to peak insulin concentration (T-peak_INS_), area under the curve for insulin (AUC_INS_) and glucose (AUC_GLU_) as well as whole body insulin sensitivity index (ISI_COMP_).

**Results:**

Compared to the healthy group, the EMS group had 6–7 times higher geometric mean for Peak_INS_ and AUC_INS_ and 8 times lower geometric mean for ISI_COMP_. The EMS group had a delayed T-peak_INS_ compared to the healthy group. There was no effect of breed in the group of healthy horses on Peak_INS_, T-peak_INS_, AUC_INS_, AUC_GLU_ and ISI_COMP_. Coefficient of variation for repeated tests was 19.8, 19.0 and 17.6 % for Peak_INS_, AUC_INS_ and ISI_COMP_ respectively.

**Conclusions:**

The results of the present study demonstrate that the modified OST appears to be a practical and useful diagnostic tool for assessment of insulin dysregulation in the horse. However, to make it possible to establish the most appropriate sampling interval and to evaluate the accuracy of the modified OST, further studies in horses with a variable degree of insulin resistance are needed, where results from the modified OST are compared with quantitative measurements for IS.

## Background

Equine metabolic syndrome (EMS) refers to a group of clinical abnormalities including generalized obesity and/or regional adiposity, insulin resistance (IR) and hyperinsulinemia [1]. Postprandial hyperinsulinemia is a risk factor for development of laminitis [2–7], and since EMS is a rising concern in the equine population worldwide, it is important to create appropriate diagnostic tests for abnormalities in insulin regulation. In humans, glucose and insulin data from an oral glucose tolerance test (OGTT) can be used to calculate surrogate estimates for insulin sensitivity (IS) that are well correlated to IS quantified by the euglycemic hyperinsulinemic clamp (EHC) [8–10]. The composite whole-body insulin sensitivity index (ISI_COMP_), also called the Matsuda index, is a commonly used OGTT-derived estimate for IS in humans [10]. The use of ISI_COMP_ as an estimate for IS in the horse has recently been evaluated against quantitative measurements of IS [11]. Despite these relationships OGTT does not measure IS per se. Instead, it is a test in which both glucose disposal and insulin secretion are stimulated but the test is influenced by factors such as gastric emptying, degree of hepatic glucose trapping, β-cell responsiveness and incretin effects [12].

Due to the association between hyperinsulinemia and laminitis in the horse [13], identification of individuals with an exaggerated postprandial insulin response using OGTT may enable better management of horses and ponies at risk for pasture-associated laminitis. An alternative to the OGTT, where glucose is used as the only carbohydrate source, is the recently developed oral sugar test (OST) [14]. This test uses a standardized amount of Karo^®^ light corn syrup, which is composed of different unknown carbohydrates. Karo^®^ light corn syrup is, however, not available in Scandinavian grocery stores. The aim of the present study was therefore to compare the results of a modified oral sugar test (OST), using a Scandinavian commercially available glucose syrup (Dansukker glykossirap), in a group of healthy horses and in horses with EMS. In addition, the effect of breed on the test result and the repeatability of the test were studied in clinically healthy horses.

## Methods

### Horses

The study was approved by the Ethical Committee for Animal Experiments, Uppsala, Sweden. Twenty-three clinically healthy horses and 20 horses with EMS were included in the study. The clinically healthy horses were seven Shetland ponies, eight Icelandic horses and eight Standardbred horses. Inclusion criteria for these horses were a body condition score (BCS) of ≤6.5 (scale 1–9) [15], a cresty neck score (CNS) of ≤3.0 (scale 0–5) [16], normal findings on clinical examination, no history of laminitis based on veterinary information and a normal plasma ACTH concentration. The Icelandic horses were used for leisure riding whereas the Standardbred horses and Shetland ponies were sedentary horses. The horses with EMS were two Fjord horses, one Swedish warmblood, ten Icelandic horses, two Shetland ponies, one Welsh Cob, one Highland pony, one Swedish riding pony, one crossbreed horse and one crossbreed pony. The horses with EMS were recruited from a group of horses referred for an extended evaluation of EMS at the University Animal hospital, Swedish University of Agricultural Sciences. Criteria for inclusion were IR, defined as a M/I ratio of ≤3.5 calculated from an EHC, and a normal plasma ACTH. In addition, horses had to have either ≥1 previous episode of laminitis not associated with other predisposing conditions or a CNS >3 [16]. Also, horses eligible to participate had no significant findings on clinical examination, were fed a roughage based diet without grain supplementation and had no ongoing episode of laminitis. Physical characteristics for the two groups of horses are reported in Table 1. All horses were privately owned except for the clinically healthy Standardbred horses, which belonged to the Swedish University of Agricultural Sciences research herd. All horses in the study were fed a hay or haylage diet supplemented with minerals and six of the Icelandic horses were feed 50–100 g of grain 3–5 times/week as a treat. Horses were housed in individual box stalls and allowed daily turnout in a dirt or a sand paddock. None of the horses had been at grass pasture for at least 2 month prior to testing.

**Table 1:** Baseline physical characteristics of horses in healthy (n = 23) and equine metabolic syndrome (EMS) (n = 20) groups

Group	Age (years)	Body weight (kg)	Body condition score (1–9)	Cresty neck score (0–5)
EMS (n = 20)	13.9 ± 4.6 (5–22)^a^	395 ± 117 (124–558)^a^	6.9 ± 1.1 (5.5–9.0)^a^	3.7 ± 0.6 (2.5–5.0)^a^
Healthy (n = 23)				
Shetland pony (n = 7)	3.9 ± 2.7 (1–9)^c^	152 ± 29 (115–194)^b^	5.6 ± 0.6 (5.0–6.5)^b^	2.6 ± 0.4 (2.0–3.0)^b^
Icelandic horse (n = 8)	7.0 ± 2.0 (5–10)^bc^	342 ± 21 (298–360)^ac^	6.0 ± 0.4 (5.5–6.5)^ab^	2.6 ± 0.2 (2.5–3.0)^b^
Standardbred horses (n = 8)	10.5 ± 4.7 (5–20)^ab^	507 ± 56 (441–595)^d^	5.0 ± 0.5 (4.5–5.5)^b^	2.0 ± 0.5 (1.5–2.5)^b^

Data are presented as mean ± standard deviation (SD) (range)

Means with different superscript letters within column differ at P < 0.02

### Experimental design

The healthy horses were examined in their home environment whereas the horses diagnosed with EMS were examined at the large animal clinic at the Swedish University of Agricultural Sciences after an acclimatization period of 2 days. All horses in the study were examined once with the modified OST. The Icelandic horses and Shetland ponies in the healthy group were examined twice with 8 days in between, to assess the repeatability of the OST. Horses in the EMS group were tested with an EHC 24 h after the OST. Both the OST and the EHC commenced after 12 h of feed withdrawal overnight but with free access to water.

### OST—oral sugar test

The day before the OST an intravenous catheter (Intranule, 2.0 × 105 mm. Vygon, Ecouen, France) was inserted aseptically into one of the jugular veins under local anesthesia (EMLA, AstraZenica AB, Södertälje, Sweden). On the morning of the OST, Dan Sukker Glykossirap (Nordic Sugar A/S, Copenhagen, Denmark) was syringed per os at a dosage of 0.2 ml/kg body weight, providing 216 mg/kg BW of saccharides (Table 2). Blood samples were collected from the jugular catheter into evacuated tubes (Vacuette 9 ml, Greiner Bio-One GmbH, Kremsmünster, Austria) containing lithium heparin, immediately before (−5 min), and at 30, 60, 90, 120, 150 and 180 min after the oral administration of syrup. After collection of each blood sample the catheter line was immediately flushed with 10 ml 0.9 % sterile saline solution. Sample tubes were centrifuged for 10 min at 2700×*g* and plasma was subsequently harvested and stored at −80 °C until analysis of plasma glucose and plasma insulin.

**Table 2:** Chemical composition of the Dan Sukker Glykossirap

Dry matter (%)	Glucose (%)	Maltose (%)	Maltotriose (%)	Other sugars (%)	Density (20 °C)
77	12	11	10	44	1.40 kg/L

Product specification | PS 236654-1.3EN. Nordic Sugar A/S, Copenhagen, Denmark

Insulin and glucose data from the OST were used to calculate the following variables: Peak insulin concentration (Peak_INS_), time to peak insulin concentration (T-peak_INS_), area under the curve for insulin (AUC_INS_), area under the curve for glucose (AUC_GLU_) and composite whole-body insulin sensitivity index (ISI_COMP_) [10]. AUC_INS_ and AUC_GLU_ were calculated by use of the trapezoid method (GraphPad Prism, version 6.0 for windows; GraphPad Software Inc, San Diego, CA). ISI_COMP_ was calculated by the following formula: ${{1000} \mathord{\left/ {\vphantom {{1000} {\sqrt {\left( {G \cdot I} \right) \cdot ( MG \cdot MI)} }}} \right. \kern-0pt} {\sqrt {\left( {G \cdot I} \right) \cdot ( MG \cdot MI)} }}$, where G = baseline glucose concentration, I = baseline insulin concentration, MG = mean glucose concentration during the OST and MI = mean insulin concentration during the OST. A numerator of 1000 was used in the formula, to transform the data into manageable values.

### EHC—euglycemic hyperinsulinemic clamp

For horses in the EMS group, a second intravenous catheter (Intranule, 2.0 × 105 mm. Vygon, Ecouen, France) was inserted into the contralateral jugular vein after the OST examination. On the morning of the following day blood samples for the determination of baseline concentrations of plasma glucose and plasma insulin were drawn from one of the intravenous catheters immediately before (−10, −5, and −1 min) the start of the EHC. The EHC technique has previously been described for use in horses [17, 18]. Briefly, a continuous rate infusion of glucose (Glucose Fresenius Kabi 500 mg/ml, Fresenius Kabi AB, Uppsala, Sweden) and recombinant human insulin (Humulin Regular, Eli Lilly Sweden AB, Solna, Sweden) was initiated through one of the jugular catheters, using a multi-channel volumetric infusion pump (Colleague, Volumetric infusion pump, Baxter Healthcare SA, Zurich, Switzerland). The infusion rate for insulin was constant at 3 mU/kg/min and a variable rate of glucose was infused to maintain blood glucose concentration at euglycemia (5 mmol/L) during the 3 h long infusion. The glucose infusion rate was adjusted if the concentration deviated by more than 0.2 mmol/L from euglycemia. Blood samples were obtained every 5 min for analysis of blood glucose using a handheld glucometer (Accu-Check Aviva, Roche Diagnostics Scandinavia AB, Bromma, Sweden) and every 10 min throughout the EHC for subsequent determination of plasma glucose (10 min intervals) and plasma insulin (20 min intervals). After collection of each blood sample, the catheter line was immediately flushed with 10 ml 0.9 % sterile saline solution.

The first 120 min of the EHC were considered an equilibration period. Glucose and insulin data from the final 60 min (steady-state) were used for calculation of insulin sensitivity (M/I ratio). The M/I ratio was calculated for each 20-min interval where M is the mean glucose infusion rate and I is the plasma insulin concentration for the interval. The M/I_60_ is the mean of the three M/I ratios during steady state [17].

### Analysis of blood samples

Plasma glucose concentrations were measured enzymatically with an automated clinical chemistry analyzer (YSI 2300 Stat Plus Analyzer, YSI Incorporated, Yellow Spring, Ohio). Endogenous equine plasma insulin concentrations from the OST were determined using a commercial equine-optimized ELISA (Mercodia Equine Insulin ELISA, Mercodia AB, Uppsala, Sweden) evaluated for use in horses [19] and insulin levels were controlled with a commercial kit (Mercodia Animal Insulin Control (Low, Medium, High), Mercodia AB, Uppsala, Sweden). Plasma insulin concentrations from the continuous rate infusion of recombinant human insulin during the EHC procedures were determined using a commercial human ELISA (Mercodia Insulin ELISA, Mercodia AB, Uppsala, Sweden) and a commercial kit (Mercodia Diabetes Antigen Control (Low, High)/Human, Mercodia AB, Uppsala, Sweden) was used as a control. All analyses of plasma glucose and insulin were performed in duplicate. The mean intra-assay CVs for glucose, equine insulin (equine-optimized ELISA) and human insulin (human ELISA) were 0.4, 2.7 and 2.7 % respectively, as determined from duplicate analyses.

### Statistical analysis

All data were analyzed in JMP^®^ Pro 11.0.0. (SAS Institute Inc., Cary, North Carolina, USA). Differences in plasma glucose and insulin concentrations for the healthy and the EMS groups were compared at different time points during the OST, by use of a 2-way ANOVA for repeated measures. Differences in physical characteristics (age, BCS, CNS and body weight) and OST derived calculated variables (AUC_GLU_, AUC_INS_, ISI_COMP_, Peak_INS_ and T-peak_INS_) were compared between breeds and between the healthy and EMS groups by use of an independent t test or a 1-way ANOVA as appropriate. Differences in calculated variables for repeated OST tests were compared by use of a paired t test. The repeatability of the OST was determined by the mean coefficient of variation (mean CV), the intraclass correlation coefficient (ICC) and the repeatability coefficient (RC). The ICC was calculated according to the formula 1−S_w_^2^/(s_b_^2^ + S_w_^2^) and the RC was calculated as 2.77S_w_, where S_w_^2^ is the within subject variance and s_b_^2^ is the between subject variance calculated from the analysis of variance table of a 1-way ANOVA [20]. Values of P < 0.05 were considered as significant for all analyses. If residuals were not normally distributed data were logarithmically transformed. Logarithmically transformed data were then expressed as the geometric mean ± 95 % CI on the original scale after back transformation. All other results were expressed as mean ± SD, whereas data for age, body weight, BCS and CNS were additionally presented with the range.

## Results

All horses included in the study fulfilled the inclusion criteria for the separate groups. All horses tolerated the administration of syrup well. The loss of syrup was negligible comprising the small amount that got stuck to the outside of the syringe during administration.

### Comparison of EMS and healthy groups

All horse in the EMS group were defined as insulin resistant by a M/I_60_ of <3.5 (μg/kg/min)/(mU/L). Fourteen of 20 included horses with EMS had a severe IR i.e. M/I_60_ < 2.0 (μg/kg/min)/(mU/L). Sixteen of the 20 horses included in the EMS group had previous episodes of laminitis. The EMS group had a higher mean CNS compared to the breeds included in the healthy group (P < 0.0004). Mean BCS was higher in the EMS group compared to the Standardbred horses and Shetland ponies of the healthy group (P < 0.010), but no difference was found between the EMS group and the healthy Icelandic horses (P = 0.074) (Table 1).

**Fig. 1:**
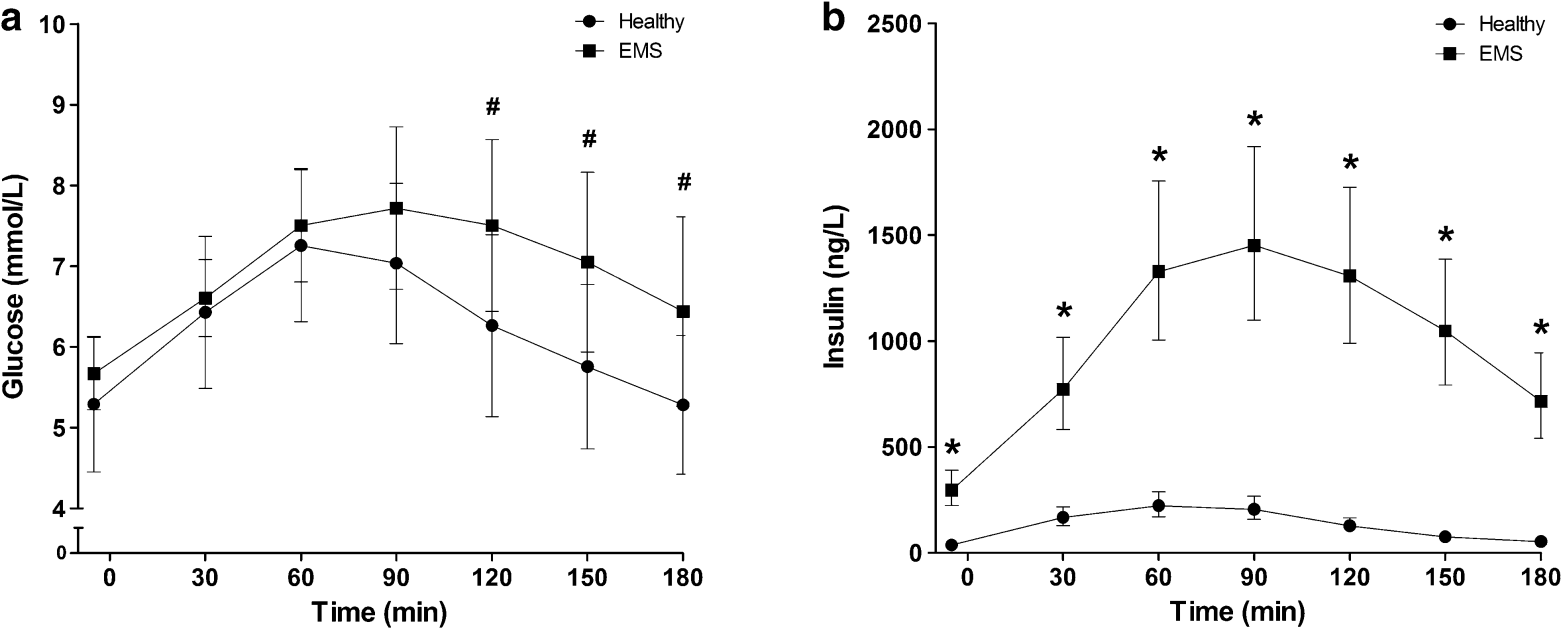
Plasma glucose (**a**) and plasma insulin (**b**) concentrations measured during the OST in healthy (n = 23) and EMS (n = 20) groups. Data are presented as mean ± SD for plasma glucose and as geometric mean, 95 % confidence interval for plasma insulin. *Groups differ at P < 0.0001. #Groups differ at P < 0.0036. *OST* oral sugar test; *EMS* equine metabolic syndrome; *SD* standard deviation

There was an overall effect of time between the group of healthy horses and EMS horses during the OST for insulin (P < 0.0001) but not for glucose (P = 0.178). An overall interaction between group of horses (clinically healthy vs EMS) and time was identified for both insulin (P < 0.0001) and glucose (P = 0.003) (Fig. 1).

**Fig. 2:**
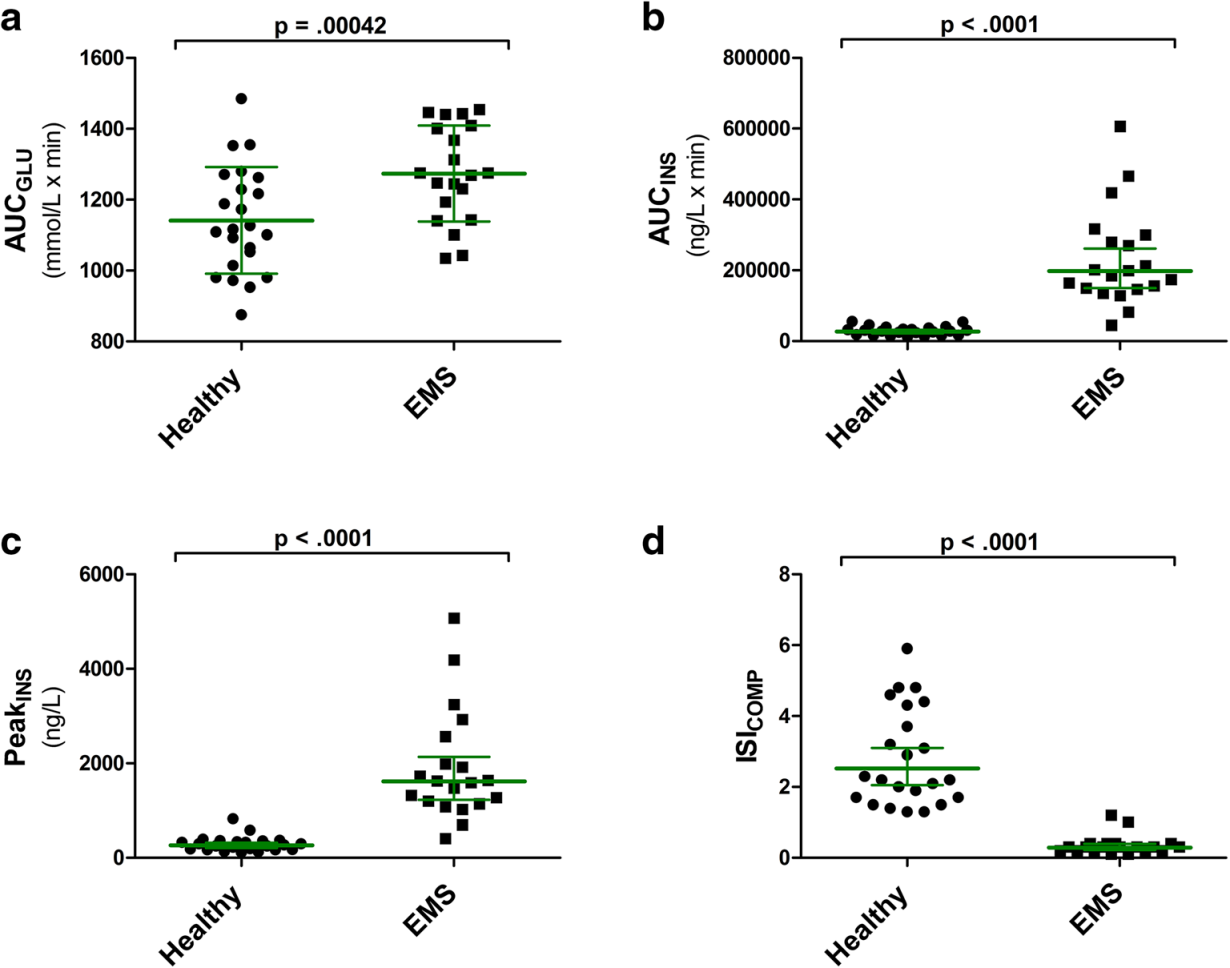
AUC_GLU_ (**a**), AUC_INS_ (**b**), Peak_INS_ (**c**) and ISI_COMP_ (**d**) values for OST performed in healthy (n = 23) and EMS (n = 20) groups. Horizontal lines present mean, SD for AUC_GLU_; and geometric mean, 95 % confidence interval for AUC_INS_, Peak_INS_ and ISI_COMP_. P < 0.05 represents statistical differences. *OST* oral sugar test; *AUC*_*GLU*_ area under the curve for glucose; *AUC*_*INS*_ area under the curve for insulin; *Peak*_*INS*_ peak concentration for insulin; *ISI*_*COMP*_ composite whole-body insulin sensitivity index; EMS, equine metabolic syndrome; *SD* standard deviation

Compared to the healthy group, the EMS group had 6–7 times higher geometric mean for Peak_INS_ (P < 0.0001) and AUC_INS_ (P < 0.0001) and 8 times lower geometric mean for ISI_COMP_ (P < 0.0001) (Fig. 2). Horses in the healthy group reached the maximum insulin concentration (T-peak_INS_) on average 69 ± 25 min from the start of the OST. Horses in the EMS group had delayed insulin peak compared to the healthy group (P = 0.007) with an average of 92 ± 27 min from the start of OST. In the healthy group geometric means (95 % CI) for insulin were 222 (172–288) ng/L and 206 (159–267) ng/L at the 60 and 90 min sampling respectively. For the EMS group the geometric means (95 % CI) for insulin for the corresponding time points were 1329 (1006–1756) ng/L and 1452 (1099–1919) ng/L, respectively.

### Breed comparison

There was no effect of breed on AUC_GLU_, AUC_INS_, Peak_INS_, ISI_COMP_ and T-peak_INS_ in the group of healthy horses (Table 3).

**Table 3:** Calculated variables and measures for the oral sugar test (OST) in healthy Shetland ponies (n = 7), Icelandic horses (n = 8) and Standardbred horses (n = 8)

	Shetland ponies	Icelandic horses	Standardbred horses	P value
AUC_GLU_ (mmol/L × min)	1071 ± 136	1239 ± 167	1100 ± 96	0.055
AUC_INS_ (ng/L × min)	32,235 ± 14,797	29,130 ± 14,655	26,967 ± 7600	0.728
ISI_COMP_	2.2 (1.5–3.2)	2.6 (1.8–3.7)	2.8 (1.9–4.0)	0.608
Peak_INS_ (ng/L)	301 (203–446)	237 (164–342)	259 (179–374)	0.652
T-peak_INS_ (min)	56 ± 27	75 ± 23	75 ± 23	0.234

Data are presented as mean ± SD for AUC_GLU_ and AUC_INS_; and as geometric mean (95 % confidence interval) for Peak_INS_ and ISI_COMP_

*AUC*_*GLU*_ area under the curve for glucose; *AUC*_*INS*_ area under the curve for insulin; *ISI*_*COMP*_ composite whole-body insulin sensitivity index; *Peak*_*INS*_ peak concentration for insulin; *T-peak*_*INS*_ time to peak concentration for insulin; *SD* standard deviation

### Repeatability testing

The AUC_GLU_, AUC_INS_, Peak_INS_, ISI_COMP_ and T-peak_INS_ did not significantly differ between repeated tests performed in the group of healthy horses (Table 4). Among the variables derived from the OST, the AUC_GLU_ had the best combination of repeatability measures i.e., a low mean CV, a high ICC and a relatively small RC. In contrast, Peak_INS_ and T-peak_INS_ had higher mean CVs as well as larger RC in relation to each of the means. Further, the Peak_INS_ had a similar ICC as AUC_GLU_, whilst T-peak_INS_ had the lowest ICC of all calculated variables.

**Table 4:** Calculated variables and measures of repeatability for the oral sugar test (OST) in 15 healthy horses (7 Shetland ponies, 8 Icelandic horses)

	Test 1	Test 2	P value	Mean CV (%)	ICC	RC
AUC_GLU_ (mmol/L × min)	1161 ± 171	1153 ± 116	0.823	6.7	0.79	237.7
AUC_INS_ (ng/L × min)	30,579 ± 14,276	31,706 ± 15,526	0.623	16.9	0.91	16,556.4
ISI_COMP_	2.7 ± 1.5	2.8 ± 1.6	0.912	17.3	0.85	2.1
Peak_INS_ (ng/L)	309 ± 190	308 ± 153	0.976	18.3	0.83	255.5
T-peak_INS_ (min)	66 ± 26	74 ± 34	0.334	18.5	0.64	60.7

Data are presented as mean ± standard deviation (SD)

*AUC*_*GLU*_ area under the curve for glucose; *AUC*_*INS*_ area under the curve for insulin; *ISI*_*COMP*_ composite whole-body insulin sensitivity index; *Peak*_*INS*_ peak concentration for insulin; *T-peak*_*INS*_ time to peak concentration for insulin; *mean CV* mean coefficient of variation; *ICC* intraclass correlation coefficient; *RC* repeatability coefficient

## Discussion

A modified OST test was developed and evaluated using a Scandinavian commercially available glucose syrup (Dan Sukker Glykossirap). Higher OST glucose and insulin plasma concentrations and accordingly lower ISI_COMP_ as well as higher AUC_GLU_, AUC_INS_ and Peak_INS_ were found in the EMS group compared to the healthy group. Moreover, the calculated parameters from the OST had moderate to high repeatability and there was no effect of breed on the test results. These results show that the modified OST appears to be a practical and useful diagnostic tool for assessment of dysinsulinemia in the horse.

It has been suggested that the OGTT and OST more closely mimic the glucose and insulin dynamics during physiological conditions than the EHC and the frequently sampled IV glucose tolerance test (FSIGTT). However, it is important to recognize that glucose and insulin responses after oral glucose delivery are not equivalent to insulin sensitivity. The glucose and insulin response after oral administration of glucose are dependent on factors such as gastric emptying, absorption, glucose uptake in the liver, incretin effects, insulin secretion and clearance as well as insulin sensitivity [12]. Thus, the OGTT and OST provide information about the efficiency of the homeostatic mechanisms including insulin-dependent mechanisms to restore blood glucose concentration to basal levels but not IS/IR per se. Nevertheless, several studies in humans, but also in the horse, have used different surrogate indices based on dynamic data from OGTT in order to estimate IS [8–11]. The most commonly used index in humans, ISI_COMP_, reflects a composite estimate of both hepatic and peripheral insulin sensitivity based on fasting glucose and insulin data as well as the mean of glucose and insulin values obtained during the OGTT. The square-root conversion in the formula is used to correct for non-linear distribution of data in relation to the IS determined by the EHC [10]. Studies in humans and horses have shown that ISI_COMP_ has a relatively good linear correlation to quantitative measurements of IS obtained by the EHC and the surrogate index therefore provides a reasonable estimate of IS [10, 11].

Compensatory hyperinsulinemia can develop in horses with IR [21, 22] by two mechanisms: increased insulin secretion from the ß-cells in the pancreas and reduced insulin clearance by the liver [21, 23–26]. This hyperinsulinemia is more pronounced and thereby easier to detect after feed intake compared to pre-prandial conditions [27, 28]. The OST is a dynamic test that mimics a feeding situation and makes it possible to study postprandial hyperinsulinemia under controlled conditions. In the present study horses in the EMS group had higher plasma insulin concentrations at all time points during the OST compared to the healthy group. The higher insulin response in the EMS group resulted in higher AUC_INS_, Peak_INS_ and lower ISI_COMP_ compared to the healthy group. In contrast, plasma glucose concentrations only differed between the healthy and the EMS group at 120, 150 and 180 min. Repeated blood sampling is necessary for calculation of AUC_INS_, AUC_GLU_ and ISI_COMP_, making these parameters less useful during field conditions. Instead, Peak_INS_ requires only a single sample, which reduces the costs for laboratory analyses as well as the time spent on sampling in the field during the test.

For the USA-developed OST [14] researchers found that peak concentrations for insulin was recorded at blood sampling at 60 or 90 min after syrup administration. Hence, the time interval between 60 and 90 min after sugar administration is recommended for single blood sampling for measurements of peak insulin concentrations. In the present study, there was a longer mean time to peak insulin in the EMS group (92 ± 27 min) compared to the healthy group (69 ± 25 min). Nevertheless, plasma insulin concentration differed between the healthy and the EMS group at all time points during the OST and there was no overlap of confidence intervals. This suggests that the time frame for single blood sampling could be extended, making the test protocol more time flexible. The largest difference in plasma insulin concentrations between the two groups of horses occurred at sampling times between 60 and 150 min. However, the majority of horses included in the EMS group of the present study had a severe IR, as determined by the EHC. Severe IR is associated with a pronounced compensatory β-cell response as well as decrease in insulin clearance [22, 25], leading to a prolonged insulin response during the OST. On the other hand, a horse in an early stage of disease with a mild to moderate degree of IR, is likely to have a shorter and less prominent insulin response. Thus, if a blood sample is taken later during the OST in these horses (for example at 150 min) the insulin concentration may then have decreased to a level seen in a normal horse. The most optimal time point for sampling in order to distinguish normal horses from those with insulin dysregulation, can therefore not be concluded from the present study since it would have required a larger number of horses with a wider range of IR and β-cell responses.

The Karo^®^ Light Corn Syrup is not available in Scandinavian grocery stores wherefore the agreement between the USA-developed OST and the modified OST could not be assessed. For the modified OST a dose of 0.2 ml/kg syrup is used, providing 216 mg saccharides/kg of body weight compared to 0.15 ml/kg of syrup for the USA developed OST that is estimated to provide the horse with 150 mg/kg of body weight of dextrose-derived digestible sugars. The total dose of sugar is thereby 44 % higher for the modified test. The glucose and insulin response after administration of syrup is not only influenced by the total amount of sugar but also the relative composition of the different sugar types. Information about the composition of the Karo^®^ Light Corn Syrup is withheld by the manufacturer, which therefore makes it difficult to design an identical test for the Scandinavian market. Another aspect that further complicates comparisons between the two tests is that different assays have been used for measurement of plasma insulin (Coat-A-Count Insulin RIA vs Mercodia Equine Insulin ELISA). Earlier performed work [19] showed that results were in good agreement between these two assays for samples with equine insulin concentrations <150 µU/ml, but for samples with higher insulin concentrations the agreement between assays was poor [29]. The calculated conversion factor for equine insulin from mU/L, determined with the radioimmunoassay (RIA) method, to the quantitative unit ng/L, determined with the equine specific ELISA is 10 [19]. The cutoff for dysinsulinemia for the USA developed OST is >60 mU/L (measured by the RIA) at sampling at 60–90 min [30, 31], corresponding to >600 ng/L for insulin concentrations measured by the equine ELISA. In the present study, 21 of the 23 horses in the healthy group had plasma insulin concentrations <400 ng/L at the 60 min sampling and at the 90 min sampling all healthy horses had plasma insulin concentrations <400 ng/L. In the EMS group 19 of 20 horses had plasma insulin concentrations >600 ng/L at 60 and 90 min. If the same sampling interval is used for the modified OST as for the USA developed OST (i.e. 60–90 min) it seems reasonable to use the same cut off values for the two tests.

No differences were found between breeds for any of the OST calculated variables (AUC_GLU_, AUC_INS_, ISI_COMP_, Peak_INS_ and T-peak_INS_). Previous studies have shown differences in IS between healthy horses of different breeds using other diagnostic tests such as the CGIT [32] or the FSIGTT [28]. A small sample in combination with a relatively large within breed variation could possibly explain why no effect of breed was found in the present study. Another aspect is that only the Icelandic horses were exercised and used for leisure riding whereas the remainder (Shetland ponies and Standardbred horses) were sedentary horses. Since exercise can affect the degree of IS [33, 34], it is possible that the outcome would have been different if the degree of activity had been similar between breeds. Another explanation might be that the test is not sensitive enough to detect small differences in IS between different breed populations.

One limitation of the present study is that one group of horses (the EMS group) was tested during hospitalization whereas all other horses were tested in their home environment. In a previous study [35] no effect of housing and diet on results from the OST were found. The EMS horses in our study were acclimatized 48 h prior to testing at the large animal clinic and horses were only fed their own hay or haylage in order to minimize the possible effect of housing and feeding. Another limitation is that normal IS evaluated by EHC was not used as inclusion criteria for the healthy group. Whereas the IS of the Standardbred horses is known to be normal based on information from previous research projects where the horses’ IS have been quantified (data not shown), the IS of the privately owned control horses is unknown. However, all control horses had normal fasting plasma insulin and glucose concentrations suggesting normal IS.

The definition of general obesity has varied among studies, e.g., BCS > 6 [5], BCS ≥ 7 [23, 36], BCS ≥ 8 [16]. Another study suggested BCS 7–7.5 as overweight but not obese [16]. The Henneke score [15] is originally adapted for use in Quarter Horses but there are no studies evaluating how well this scoring system agrees between different breeds. In addition, there seems to be breed variations as well as seasonal variations in CNS [37]. There is thus no consensus on how overweight or abnormal local adiposity should be defined for different breeds of horses. It is possible that the inclusion criteria for BCS and CNS among the normal horses in the present study should have used a narrower range. To test the hypothesis that BCS or CNS among the normal horses were associated with fasting insulin concentrations, Peak_INS_ or ISI_COMP_ linear regressions were performed. These regression models showed no significant correlations (P > 0.160, data not shown) suggesting no effect of BCS or CNS on the insulin response or estimates for IS in the normal horses.

The CV values for repeated tests in the present study varied between 6.7 and 18.5 % for calculated variables, where AUC_GLU_ showed the lowest variation between tests. Higher intra-assay variability was found for analysis of insulin than for glucose in the present study, possibly affecting the CV values for the calculated parameters. In comparison, a previous study [14] using the USA developed OST, reported a similar CV for AUC_GLU_ (6.4 %) but a higher CV for the AUC_INS_ (45.1 %). One reason for the discrepancy of results is that fewer horses were used for repeatability testing in the aforementioned study. The insulin derived variables from the modified OST were generally considered to have a moderate to high repeatability based on CV, ICC and RC. Thus, repeatability of the OST appears to be comparable to the calculated variables from the insulin curve of another non-quantitative dynamic tests, the CGIT [32].

## Conclusions

The results of the present study demonstrate that the modified OST appears to be a practical and useful diagnostic tool for assessment of dysinsulinemia in the horse. However, to make it possible to establish the most appropriate sampling interval and to evaluate the accuracy of the modified OST, further studies in horses with a variable degree of insulin resistance are needed, where results from the modified OST are compared with quantitative measurements for IS.

## Abbreviations

AUC_INS_: area under the curve for insulin during OST
AUC_GLU_: area under the curve for glucose during OST
BCS: body condition score
CNS: cresty neck score
CV: coefficient of variation
EHC: euglycemic hyperinsulinemic clamp
EMS: equine metabolic syndrome
FSIGTT: frequently sampled IV glucose tolerance test
ICC: intraclass correlation coefficient
IS: insulin sensitivity
ISI_COMP_: composite whole-body insulin sensitivity index
IR: insulin resistance
M/I_60_: mean rate of glucose disposal per unit of insulin during the last 60 min of the
OGTT: oral glucose tolerance test
OST: oral sugar test
Peak_INS_: peak concentration of insulin during OST
RC: repeatability coefficient
SD: standard deviation
T-peak_INS_: time (min) to peak concentration for insulin during OST
